# Chromosomal microarray analysis in a cohort of underrepresented population identifies *SERINC2* as a novel candidate gene for autism spectrum disorder

**DOI:** 10.1038/s41598-017-12317-3

**Published:** 2017-09-21

**Authors:** Areerat Hnoonual, Weerin Thammachote, Thipwimol Tim-Aroon, Kitiwan Rojnueangnit, Tippawan Hansakunachai, Tasanawat Sombuntham, Rawiwan Roongpraiwan, Juthamas Worachotekamjorn, Jariya Chuthapisith, Suthat Fucharoen, Duangrurdee Wattanasirichaigoon, Nichara Ruangdaraganon, Pornprot Limprasert, Natini Jinawath

**Affiliations:** 1 Graduate Program in Biomedical Sciences, Prince of Songkla University, Songkhla, Thailand; 2 Program in Translational Medicine, Faculty of Medicine Ramathibodi Hospital, Mahidol University, Bangkok, Thailand; 3 Division of Medical Genetics, Department of Pediatrics, Faculty of Medicine Ramathibodi Hospital, Mahidol University, Bangkok, Thailand; 4 Division of Medical Genetics, Department of Pediatrics, Faculty of Medicine, Thammasart University, Pathumthani, Thailand; 5 Division of Child Development, Department of Pediatrics, Faculty of Medicine, Thammasart University, Pathumthani, Thailand; 6 Division of Developmental-Behavioral Pediatrics, Department of Pediatrics, Faculty of Medicine Ramathibodi Hospital, Mahidol University, Bangkok, Thailand; 7 Division of Child Development, Department of Pediatrics, Faculty of Medicine, Prince of Songkla University, Songkhla, Thailand; 8 Thalassemia Research Center, Institute of Molecular Biosciences, Mahidol University, Salaya, Nakhon Pathom Thailand; 9 Division of Human Genetics, Department of Pathology, Faculty of Medicine, Prince of Songkla University, Songkhla, Thailand; 10 Integrative Computational Bioscience Center, Mahidol University, Salaya, Nakhon Pathom Thailand

## Abstract

Chromosomal microarray (CMA) is now recognized as the first-tier genetic test for detection of copy number variations (CNVs) in patients with autism spectrum disorder (ASD). The aims of this study were to identify known and novel ASD associated-CNVs and to evaluate the diagnostic yield of CMA in Thai patients with ASD. The Infinium CytoSNP-850K BeadChip was used to detect CNVs in 114 Thai patients comprised of 68 retrospective ASD patients (group 1) with the use of CMA as a second line test and 46 prospective ASD and developmental delay patients (group 2) with the use of CMA as the first-tier test. We identified 7 (6.1%) pathogenic CNVs and 22 (19.3%) variants of uncertain clinical significance (VOUS). A total of 29 patients with pathogenic CNVs and VOUS were found in 22% (15/68) and 30.4% (14/46) of the patients in groups 1 and 2, respectively. The difference in detected CNV frequencies between the 2 groups was not statistically significant (Chi square = 1.02, df = 1, *P* = 0.31). In addition, we propose one novel ASD candidate gene, *SERINC2*, which warrants further investigation. Our findings provide supportive evidence that CMA studies using population-specific reference databases in underrepresented populations are useful for identification of novel candidate genes.

## Introduction

Autism spectrum disorder (ASD) is a complex neurodevelopmental disorder characterized by impairments in social interaction and communication, as well as stereotyped behaviors and restricted interests. For approximately 80% of cases, the cause of ASD is still unknown. Due to clinical diagnostic criteria of ASD with broader spectrum of symptoms, the prevalence of ASD is increasing worldwide. The estimated ASD prevalence in the US was 1 in 150 children in 2000, but by 2012 that had increased to 1 in 68 children^1^. In Thailand, the incidence rate of severe ASD was estimated at 9.9 per 10,000 in children aged 1–5 years^2^. Up to 70% of individuals with ASD also have intellectual disability/developmental delay (ID/DD). Genetic causes, including chromosomal abnormalities, copy number variations (CNVs), and single nucleotide variants are recognized as causative in 10–20% of ASD cases^3^. Knowing the genetic cause of ASD can help with genetic counseling and evaluation of recurrence risk in the families.

Since 2010, chromosomal microarray (CMA) has been recommended as the first-tier clinical diagnostic test for detection of CNVs in patients with ASD, ID, DD and multiple congenital abnormalities (MCA) of unknown causes^4,5^. CMA has a much higher diagnostic yield (10–20%) for these individuals than conventional cytogenetics (~3%)^5–7^. Most of CMA studies in these disorders have largely focused on Caucasian populations^4,8–26^. To date only a few non-Caucasian studies have been reported^27–32^. In addition, CMA studies in specific populations have been shown to help widen the list of novel ASD candidate genes such as *PTDSS1* and *AGER* as novel candidate genes of ASD in Lebanese subjects^31^, and population-specific CNVs in the *YWHAE* gene in Han Chinese subjects^33^. In our earlier study, we have categorized reference CNVs of more than 3,000 Thai individuals and created a CNV database that would facilitate the accurate clinical CNV interpretation for Thai patients^34^. In this study, we aimed to screen and identify known and novel CNVs associated with ASD in a large cohort of Thai patients with ASD. This is the first report demonstrating the utility of CMA for detection of CNVs in Thai patients referred with ASD of unknown cause. Our findings also provide supportive evidence that CMA studies in underrepresented populations can be useful for identification of novel candidate genes.

## Results

### Diagnostic yield of CMA in Thai patients with ASD

**Figure 1:**
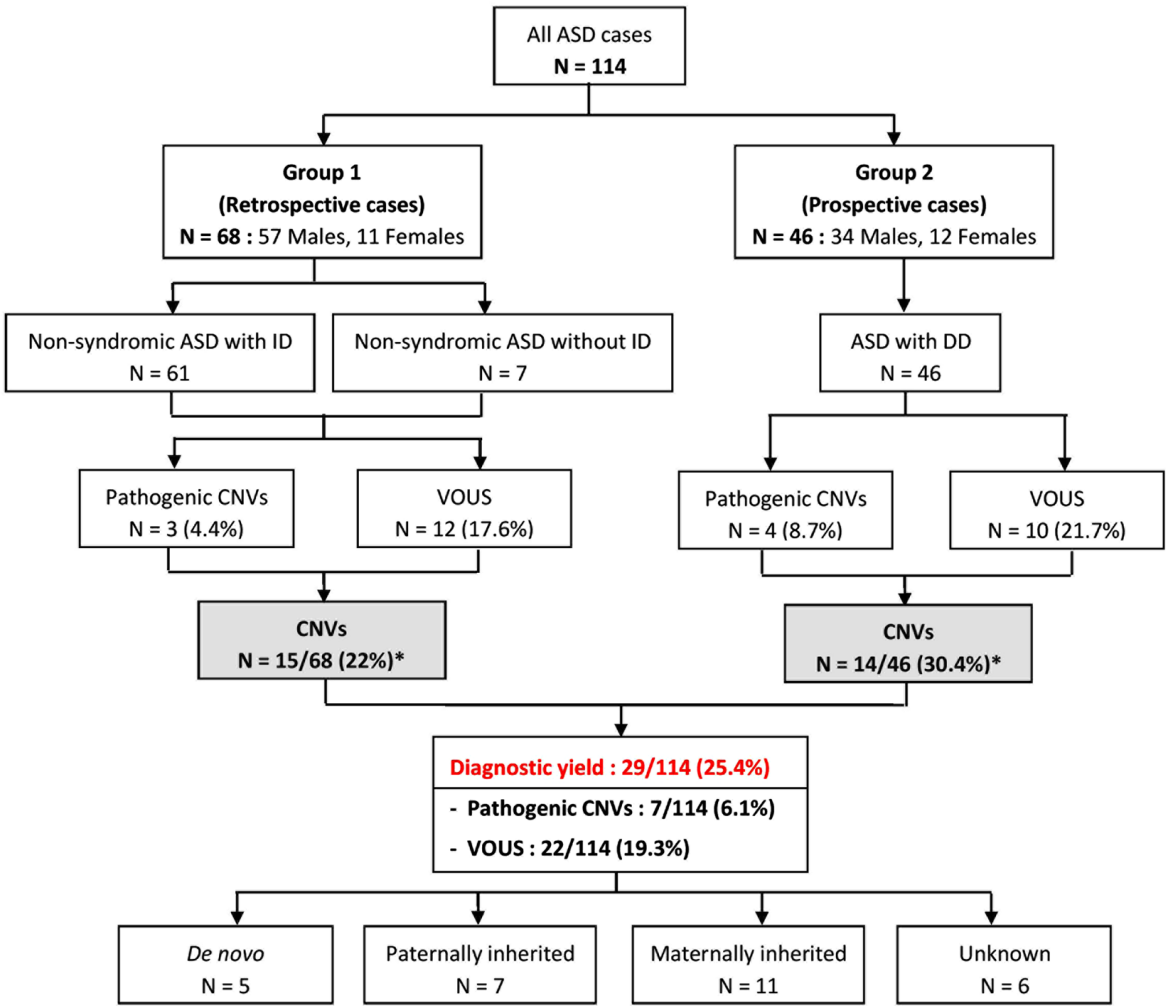
A Flowchart showing the patient characteristics and CMA analysis results in this study. *No statistically significant difference in frequencies of diagnostic yields between the 2 groups were observed using Chi-square test, *P* > 0.05 (Chi-squared = 1.02, df = 1, *P* = 0.31).

**Figure 2:**
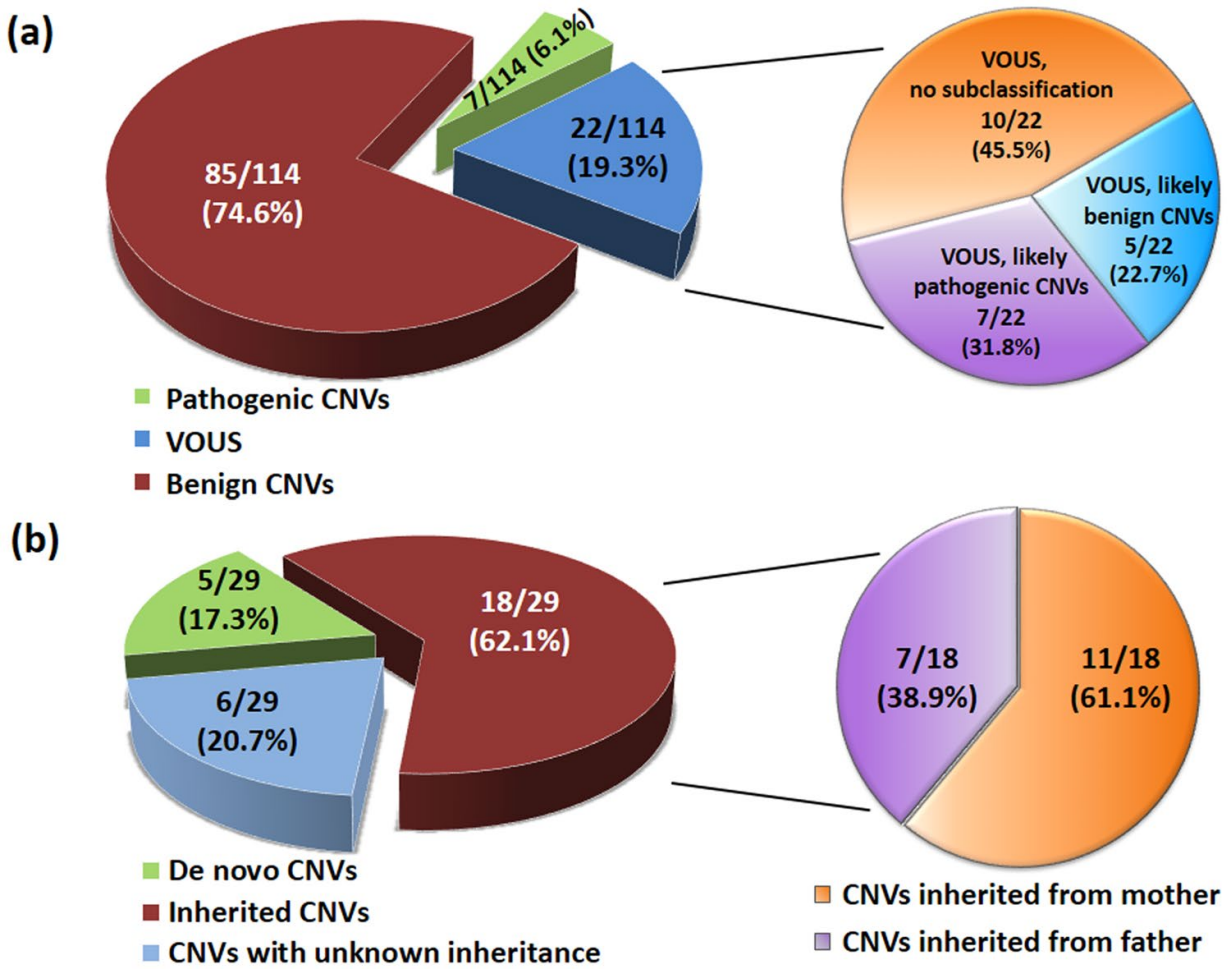
Pie charts summarizing the CNV classifications from the study. (**a**) Number of pathogenic CNVs and VOUS (likely pathogenic CNVs, likely benign CNVs, and VOUS with no subclassification); (**b**) Number of paternally or maternally inherited CNVs and *de novo* CNVs.

In our cohort of 114 Thai patients with ASD, a total of 742 CNVs was identified from all patients, ranging between 1 to 22 CNVs per patient. The size of the CNVs ranged from 1,632 bp to 18.9 Mb, with a median size of 86 kb. Among these CNVs, pathogenic CNVs and VOUS were identified in 7 (6.1%) and 22 (19.3%) patients, respectively. The overall diagnostic yield of CNVs in our cohort was 25.4% (29/114). The 29 patients with pathogenic CNVs and VOUS included 15 of 68 (22%) patients in group 1 and 14 of 46 (30.4%) patients in group 2. The summary and schematic diagrams of CMA results in the study are shown in Table 1, Figs 1 and 2. Although a higher frequency of both pathogenic CNVs and VOUS were identified in group 2 patients (30.4%) with the use of CMA as the first-tier test compared with patients in group 1 (22%) with the use of CMA as the second line test, no statistically significant differences were found in the frequencies between the 2 groups (Chi square = 1.02, df = 1, *P* = 0.31).

**Table 1:** Comparison of clinical CNVs identified in the two groups of our ASD subjects.

	**Group 1** (**Retrospective cases**)	**Group 2** (**Prospective cases**)	**Total**	**Chi squared test** (***Ρ -*** **value**)*
	**N = 68**	**N = 46**	**N = 114**	
Pathogenic CNVs and VOUS	15/68 (22%)	14/46 (30.4%)	29/114 (25.4%)	0.31
Pathogenic CNVs and VOUS, likely pathogenic CNVs	9/68 (13.2%)	5/46 (10.9%)	14/114 (12.3%)	0.71
**Pathogenic CNVs**	3/68 (4.4%)	4/46 (8.7%)	7/114 (6.1%)	0.35
Deletion	1	3	4	—
Duplication	2	1	3	—
**VOUS**	12/68 (17.6%)	10/46 (21.7%)	22/114 (19.3%)	0.59
VOUS, likely pathogenic CNVs	6/68 (8.8%)	1/46 (2.2%)	7/114 (6.1%)	0.15
Deletion	2	1	3	—
Duplication	4	0	4	—
VOUS, likely benign CNVs	3/68 (4.4%)	2/46 (4.3%)	5/114 (4.4%)	0.99
Deletion	2	1	3	
Duplication	1	1	2	
VOUS (no subclassification)	3/68 (4.4%)	7/46 (15.2%)	10/114 (8.7%)	0.04
Deletion	1	2	3	—
Duplication	2	5	7	—
**Benign**	53/68 (78%)	32/46 (69.6%)	85/114 (74.6%)	0.31

The group 1 patients were first evaluated for the common genetic causes of ASD and all patients had normal karyotype and negative DNA tests for Fragile X syndrome and MECP2 mutations. Therefore, group 1 represented the ASD patients with the use of CMA as a second line test and group 2 represented the ASD patients with the use of CMA as the first-tier test. VOUS, Variant of uncertain clinical significance**;** *P-values were calculated using Chi-square test using GraphPad QuickCalcs (https://graphpad.com/quickcalcs/contingency1.cfm).

Parental testings were available for investigation of the inheritance status in 23 of 29 patients. Of these 23 patients, 5 *de novo* CNVs, 11 maternally inherited CNVs and 7 paternally inherited CNVs were identified (Fig. 2b). However, parental samples were not available in 3 pathogenic CNVs and 3 VOUS which was a limitation of our study. The clinical characteristics of patients and details of the pathogenic CNVs and VOUS are summarized in Table 2

**Table 2:** Summary of all pathogenic CNVs and VOUS identified in this study.

Case ID	Sex	Clinical diagnosis	Additional clinical features	Chromosome region	Coordinates (hg19)	Gain/loss	Size	Gene involved*	Inheritance
**A**. **Pathogenic CNVs** (**N = 7 of 114**)									
AR82-3	M	Non-syndromic ASD	Macrocephaly	1q21.1-1q21.2	chr1:146571244-147825548	Gain	1.25 Mb	9 OMIM genes	Maternal
TU22	M	ASD, ID	Microcephaly, seizures	4p16.3	chr4:49450-1997786	Loss	1.95 Mb	22 OMIM genes	NA
2715	M	ASD, ID	Dysmorphic features, seizures	9q21.11-9q21.2	chr9:72066646–79948984	Loss	7.88 Mb	22 OMIM genes	*De novo*
AR83-3	F	Non-syndromic ASD	None	15q13.2-15q13.3	chr15:30371774-32514341	Loss	2.14 Mb	8 OMIM genes	Maternal
TM50-3	M	Non-syndromic ASD	None	16p13.11	chr16:15129970-16633361	Gain	1.50 Mb	11 OMIM genes	Paternal
TU17	F	ASD, ID, MCA	Microcephaly, cleft lip and palate, absent philtrum, abnormal ear canal, hypotelorism, flat nasal root, prominent nose	18q21.33-18q23	chr18:59030666-78015180	Loss	18.9 Mb	43 OMIM genes	NA
2950	F	ASD	Familial history of lissencephaly	22q13.33	chr22:50537901-51211392	Loss	673 kb	23 OMIM genes	NA
*OMIM genes are shown in Supplementary Table S1.									
**B**. **VOUS**, **likely pathogenic CNVs** (**N = 7 of 114**)									
TM41-3	M	Non-syndromic ASD	None	1p35.2	chr1:31707971-31928387	Gain	220 kb	*FABP3*, *SNRNP40*, *NKAIN1*,*SERINC2*	*De novo*
AR44-3	M	Non-syndromic ASD	Macrocephaly	1q42.2	chr1:231763496-231877073	Loss	113 kb	*DISC1*	NA
TM18-3	F	Non-syndromic ASD	None	2q14.1	chr2:116,548,326-116,675,350	Gain	127 kb	*DPP10*	*De novo*
TM4-3	M	Non-syndromic ASD	Macrocephaly	3p26.3	chr3:104972-264293	Gain	159 kb	*CHL1*	*De novo*
PS12-3	F	Non-syndromic ASD	None	7q31.1	chr7:111080241-111183610	Loss	103 kb	*IMMP2L*	NA
RA29	M	ASD, ID	None	15q13.3	chr15:32,389,362-32,415,088	Loss	25 kb	*CHRNA7*	NA (Not paternal)
AR12-3	M	Non-syndromic ASD	Abnormal hearing test	18q22.3	chr18:72186404-72404396	Gain	217 kb	*ZNF407*, *CNDP2*, *CNDP1*	*De novo*
**C**. **VOUS**, **likely benign CNVs** (**N = 5 of 114**)									
TM13-3	M	Non-syndromic ASD	Macrocephaly	4q24	chr4:103560963-103658350	Loss	97 kb	*MANBA*	Paternal
PS37-3	M	Non-syndromic ASD	seizures	10q23.1	chr10:84017608-84115017	Loss	97 kb	*NRG3* (*intronic*)	Maternal
AR33-3	M	Non-syndromic ASD	None	17p13.3	chr17:228978-440497	Gain	211 kb	*FAM101B*, *VPS53*	Paternal
RA11	M	ASD, ID	None	Xp11.4	chrX:41252835-41363836	Gain	111 kb	*NYX*, (near *CASK*)	Maternal
RA32	M	ASD, ID	None	Xq28	chrX:148086944-148291691	Loss	204 kb	No gene (near *AFF2*)	Maternal
**D**. **VOUS** (**no subclassification**) (**N = 10 of 114**)									
AR91-3	M	Non-syndromic ASD	None	2q13	chr2:112581082-112858485	Gain	277 kb	*ANAPC1*, *MERTK*	Maternal
RA15	M	ASD, ID	None	2q12.2-2q12.3	chr2:106637427-107642482	Gain	1.0 Mb	*C2orf40*, *USX1*, *PLGLA*, *RGPD3*, *ST6GAL2*	Maternal
TM35-3	F	Non-syndromic ASD	None	4q28.2	chr4:128901402-130163564	Gain	1.26 Mb	*SCLT1*, *PHF17*, *PGRMC2*	Paternal
2546	M	ASD, GDD, MCA	Generalized hypotonia, clenched hands, microphthalmia, large ear	7q11.22	chr7:69718336-69783020	Loss	64 kb	*AUTS2*	Maternal
TM54-3	M	Non-syndromic ASD	None	8q24.3	chr8:142,039,892-143,535,189	Loss	1.49 Mb	*PTP4A3*, *GPR20*	Paternal
TU29	F	ASD, ID	Facial dysmorphism, seizures	15q26.2	chr15:95048523-96519797	Gain	1.47 Mb	Reference genes: *LOC440311*, *LINC00924*	Maternal
TU23	F	ASD, ID MCA	Microcephaly, brain abnormalities, facial dysmorphism, congenital heart defect, abnormal hearing test, congenital cataract	18q21.31-8q21.32	chr18:55411917-56384192	Gain	972 kb	*ATP8B1*, *NEDD4L*, *MIRN122A*, *MALT1*	Paternal
RA39	M	ASD, ID	None	20p12.1	chr20:14562027-14714457	Loss	152 kb	*MACROD2*	Paternal
TU4	M	Non-syndromic ASD	Short philtrum, 2 hair whirls, small eyes	Xp22.31	chrX:6449601-8141017	Gain	1.69 Mb	*HDHD1A*, *STS*, *VCX*, *VCX2*, *VCX3 A*, *PNPLA4*	Maternal
RA6	M	ASD, ID	None	Xq28	chrX:154735698-155094135	Gain	358 kb	*TMLHE*, *SPRY3*	Maternal

Abbreviations: VOUS, variant of uncertain clinical significance; ASD, autism spectrum disorder; ID, intellectual disability; DD, developmental delay; GDD, global developmental delay; MCA, multiple congenital anomalies; NA, not available.

.

### Rare inherited CNVs and known syndromes

18 of the 23 CNVs in this study were transmitted from an asymptomatic parent. Most of the VOUS with inherited CNVs were below 1 Mb, but five CNVs were more than 1 Mb in size including four duplications and one deletion. Interestingly, one (TM54–3) was a 1.49 Mb deletion encompassing 2 OMIM genes (*PTP4A3*, *GPR20*), which was inherited from an asymptomatic father. Deletions in these regions have been reported as both pathogenic CNVs and VOUS in the ISCA/ClinGen and DECIPHER databases. Thus, it is difficult to interpret the significance of the deletion in this patient. However, we assumed that it was a VOUS, because genes in this region were not involved in neurological function which may have been associated with the phenotype of this patient.

Within the group of patients with pathogenic CNVs, there were patients with well-known microduplication or microdeletion syndromes, including the 1q21.1 duplication, 15q13.3 microdeletion, 16p13.11 microduplication, 22q13.3 deletion (Phelan-McDermid syndrome), 18q distal deletion, and 4p16.3 distal deletion (Wolf-Hirschhorn syndrome). Three of the pathogenic CNVs were inherited from a healthy parent. This is in line with the fact that some of these established syndromes are known to have incomplete penetrance and/or variable expressivity.

### *De novo* CNVs and novel candidate genes for ASD

Among the *de novo* CNVs, one was a large deletion associated with the known 9q21.13 microdeletion syndrome, while the other 4 *de novo* VOUS were rare duplications of 127–220 Kb in size. A 127 kb duplication at 2q14.1 overlapping the *DPP10* gene was identified in a female proband (TM18-3) and a 159 kb duplication at 3p26.3 overlapping *CHL1* gene was identified in a male proband (TM4-3). Both the *DPP10* and *CHL1* genes have been previously reported as candidate genes of ASD. One male patient with non-syndromic ASD (TM41-3) had a 220 kb duplication at 1p35.2 including the *FABP3*, *SNRNP40*, *NKAIN1* and *SERINC2* genes. A *de novo* 217 kb duplication at 18q22.3 including the *ZNF407*, *CNDP2* and *CNDP1* genes was identified in a male proband with non-syndromic ASD (AR12-3). Table 3 provides details of all *de novo* CNVs and the mapping of the *de novo* duplications on chromosomes 1p35.2 and 18q22.3 in multiple CNV databases is shown in Supplementary Figure 1.

**Table 3:** *De novo* CNVs identified in this study.

Case ID	Chromosome region	Type of CNV	Gene involved	Gene functions and supportive evidence
2715	9q21.11-9q21.2	7.88 Mb loss	22 OMIM genes	9q21.13 microdeletion syndrome including *RORB*, which is a strong candidate gene for neurological disorders^82^.
TM41-3	1p35.2	220 kb gain	*FABP3*	*- FABP3* is a candidate tumor suppressor gene for human breast cancer and plays a role in mammary gland differentiation.
				- *FABP3* knockout mice showed decreased social memory and novelty seeking^58^.
			*SNRNP40*	*SNRNP40* is a component of the U5 small nuclear ribonucleoprotein (snRNP) particle which is involved in the removal of introns from nuclear pre-mRNAs^83^.
			*NKAIN1*	*- NKAIN1* can interact with the β subunit of Na, K-ATPase.
				- NKAIN1 protein highly expressed in hippocampus and cerebellar granular cell layer^59^.
			***SERINC2***	- *SERINC2* encodes transmembrane proteins that facilitate incorporation of serine into membrane lipid synthesis. SERINC2 mRNA was upregulated by kainate-induced seizures in the dentate gyrus of the hippocampus in the rat brain^60^.
TM18-3	2q14.1	127 kb gain	*DPP10*	*- DPP10* encodes a protein which binds specific voltage-gated potassium channels (*KCND2*) and alters their expression and biophysical properties^84^.
				- Loss and gain of the *DPP10* gene have been identified in patients with autism^49^.
TM4-3	3p26.3	159 kb gain	*CHL1*	*- CHL1* encodes a member of the L1 family of neural cell adhesion molecules, which plays a role in nervous system development and synaptic plasticity^85,86^.
				- Loss and gain of the *CHL1* gene have been identified in patients with autism and ID^50,51^.
AR12-3	18q22.3	217 kb gain	*ZNF407*	*- ZNF407* encodes a zinc finger protein which may be involved in transcriptional regulation.
				- Balanced translocation and point mutations in the *ZNF407* gene have been identified in ID patients with autism^52^.
			*CNDP2*	*CNDP2* is a nonspecific dipeptidase rather than a selective carnosinase^53^.
			***CNDP1***	*- CNDP1* protein present in pyramidal neurons of the hippocampus and neurons of the temporal cortex^53^.
				- Decreased serum *CNDP1* activity has been associated with ID, DD, multiple sclerosis and Parkinson’s disease^54–57^.

Abbreviations: ID, intellectual disability; DD, developmental delay.

### Incidental findings of common α-thalassemia mutations

In this study, we identified the deletions of α-globin genes, *HBA1* and *HBA2* as the most common incidental findings in our patient cohort. Chromosomal microarray was able to detect the common deletions of *HBA1* and *HBA2*, leading to the identification of α-thalassemia mutations in 9 of the 114 ASD patients (7.89%). These α-thalassemia deletions were confirmed by single-tube multiplex-PCR assay as previously described^35^. Among the 9 patients, eight had available DNA for validation. The results confirmed 5 cases with heterozygous alpha-thalassemia-1 (–^SEA^/αα), one case with heterozygous alpha-thalassemia-2 (-α^3.7^/αα), one case with homozygous alpha-thalassemia-2 (-α^3.7^/-α^3.7^) and the other case with compound heterozygous alpha-thalassemia-1 and alpha-thalassemia-2 (-^SEA^ /- α^4.2^), also known as Hb H disease (Supplementary Table S2).

### Detection of absence of heterozygosity (AOH) regions

We also analyzed the AOH regions greater than 10 Mb in size in all patients. One case showed two large AOH regions of 40.6 Mb (15q11.1-15q22.2) and 12.4 Mb (15q26.1-15q26.3) interrupted by a region of heterozygosity (Supplementary Figure 2), a condition which was suspected to be from uniparental disomy (UPD). Methylation-specific polymerase chain reaction (MS-PCR) analysis of *SNRPN* gene was then performed using primers as previously described^36,37^, and the results confirmed that this patient had maternal uniparental isodisomy of the 15q11.2 region resulting in Prader-Willi syndrome (Supplementary Figure 3). Additionally, multiple large AOH regions (average 158.02 Mb) were also detected in another patient, encompassing 5.84% of the genome (data not shown), suggesting a consanguineous marriage between first cousin parents (coefficient of inbreeding (F) 1/16). Since this patient had an increased chance of having an autosomal recessive disease, it might be beneficial to further search for homozygous gene mutations by means of whole exome sequencing.

## Discussion

We performed CMA in 114 Thai patients with ASD of unknown cause, resulting in an overall diagnostic yield of 25.4% composed of both pathogenic CNVs and VOUS. The diagnostic yields of only pathogenic CNVs and of VOUS alone are 6.1% and 19.3%, respectively. Our pathogenic CNV’s diagnostic yield is in line with those of other CMA studies in non-Asian patients with ASD, which range from 5.4% to 8.6%^9,12,18,22^. However, it is difficult to directly compare such diagnostic yields among studies due to the use of different inclusion criteria for diagnostic yields. Some studies report diagnostic yields from only pathogenic CNVs^4,8,11,13–18,20,22,24,26,28,29,32^, while others include combined pathogenic CNVs and likely pathogenic CNVs^10,23,25,30^ or combined pathogenic CNVs and VOUS^12,19,21,27^. In addition, previous studies have had different inclusion criteria for participating patients and used various microarray platforms with different resolutions. A summary of microarray studies in patients with ASD is given in Supplementary Table S3. Of note, because the diagnostic yield of karyotype abnormalities in ASD patients has been shown to be lower than 3%^7,23^, it is not surprising that there were no statistically significant differences in the detected frequencies of pathogenic CNVs and VOUS between group 1 patients with normal karyotype and group 2 patients with the use of CMA as the first-tier test. Therefore, our findings support the recommendation of using CMA as the first-tier diagnostic test in patients with ASD of unknown cause.

Among pathogenic CNVs, the16p13.11 microduplication was initially considered as a rare benign CNV^38^, however, we interpreted this duplication as pathogenic because several previous studies have supported the positive association between this duplication and a variety of neuropsychiatric disorders including ASD, unexplained ID, schizophrenia, epilepsy, and attention-deficit hyperactivity disorder (ADHD). This duplication region contains two strong candidate genes, *NTAN1* and *NDE1*, which are both expressed in the brain and have been proposed as candidate genes for ASD and other neuropsychiatric disorders^39–42^. Most of the pathogenic, likely pathogenic CNVs and VOUS identified in this study were transmitted from a healthy parent (18 cases from 23 families, from which parental DNA samples were available). These inherited CNVs may suggest incomplete penetrance or some other factors interacting with the CNVs. Likewise, pathogenic CNVs in 1q21.1-1q21.2 (duplication)^43–45^, 15q13.2-15q13.3 (deletion)^46,47^, and 16p13.11 (duplication)^40,41^ were also found to be inherited in our study. The other possible theory would be the two-hit model, i.e. a second mutation not detected by CMA^48^. Our findings further emphasize that parental array study alone may not be conclusive enough for CNV interpretation, the VOUS found in microarray studies need to be further analyzed before making a definite conclusion as to whether they are pathogenic or benign CNVs.

In the five *de novo* CNVs, one was a large deletion associated with the known 9q21.13 microdeletion syndrome, while the other four were duplications ranging from 127–220 kb in size. These duplications contained recently identified ASD-associated genes, *DPP10* ^49^ and *CHL1* ^50,51^, and two genes that are highly expressed in the brain, but have not yet been linked to ASD, namely carnosine dipeptidase 1 (*CNDP1*) and serine incorporator 2 (*SERINC2*). Duplications of neither *SERINC2* nor *CNDP1* have been reported in the Thai CNV database^34^. The 1p35.2 duplication containing *SERINC2* in particular is rare, and is not seen in the DGV database. Loss-of-function (LoF) variants (nonsense, frameshift and splice site variants) of these two genes were not observed in the 195 unrelated Thais from our in-house exome sequencing database (data not shown). In addition, 16 rare LoF variants of *SERINC2* (MAF < 0.001) and 27 rare LoF variants of *CNDP1* (MAF < 0.001) were found in the Exome Aggregation Consortium (ExAC) database. However, clinical phenotype data was not available for these individuals.

A *de novo* duplication of chromosome 18q22.3 included the *ZNF407*, *CNDP2* and *CNDP1* genes. Balanced translocation and point mutations in the *ZNF407* gene were identified in one study of ID patients with autism^52^, but deletions/duplications of *ZNF407* and *CNDP2* genes have not been previously reported in patients with autism. The Carnosine dipeptidase 1 gene (*CNDP1*, OMIM 609064), also known as serum carnosinase (*CN1*), is involved in neuroprotective actions and neurotransmitter functions in the brain. It is mainly expressed in the cytosol of pyramidal neurons in the hippocampus and in large and small neurons of the temporal cortex in the human brain^53^. Alterations of serum carnosinase expression and activity have been shown to be associated with neurological diseases including multiple sclerosis and Parkinson’s disease^54^. Although carnosinase deficiency has been reported in patients with ID and DD^55–57^, the overexpression of *CNDP1* from copy number gain as seen in our patient has never, to our knowledge, been previously reported. Additionally, carnosinase is an enzyme whose alterations is likely to be recessively inherited, so *CNDP1* gene duplication may not affect the carnosinase function. A search of the DGV database found no CNVs which encompassed the exact same duplicated region, but we did find several smaller duplications encompassing *CNDP1* in the general population. A search of the DECIPHER database did not reveal cases with deletion and duplication relatively similar in size to this duplication region. In addition, two DD cases (nssv581777 and nssv707115) with 178.39 and 279.02 kb duplications of the *CNDP1* region were submitted to the ISCA/ClinGen database and interpreted as benign and uncertain-likely benign (Supplementary Figure 1a). Thus, these findings do not support *CNDP1* as an ASD candidate gene.

A *de novo* 220 kb duplication of chromosome 1p35.2 encompassed the *FABP3*, *SNRNP40*, *NKAIN1* and *SERINC2* genes. Although *FABP3* knockout mice have shown decreased social memory and novelty seeking^58^ and one study found the NKAIN1 protein strongly expressed in the hippocampus and cerebellar granular cell layer^59^, no evidence for association between duplication of *FABP3*, *SNRNP40*, and *NKAIN1* genes and neurodevelopmental disorders has been reported. Of particular interest is the serine incorporator 2 gene (*SERINC2*, OMIM 614549), also known as tumor differentially expressed 2-like (*TDE2*), which encodes a transmembrane protein that facilitates incorporation of serine into phosphatidylserine and sphingolipids^60^. The concentration of sphingolipids is highest in the brain, they play important roles in neural plasticity, signaling and axonal guidance^61^. Expression of *Serinc2* mRNA was upregulated in the dentate gyrus of the hippocampus and the cerebellar Purkinje cell layer following kainate-induced seizures in rat^60^. In addition, overexpressed SERINC2 protein was reported in patients with developmental delay carrying deletions or mutations of the *UPF2 or UPF3B* genes, both of which are implicated in nonsense-mediated mRNA decay pathway (NMD)^62^. Furthermore, rare *SERINC2* variants were significantly associated with alcohol dependence in subjects of European descent^63^. Transcript expressions of genes in the *NKAIN1-SERINC2* genomic region were significantly associated with expressions of genes in the dopaminergic, serotoninergic, cholinergic, GABAergic, glutamatergic, histaminergic, endocannabinoid, metabolic, neuropeptide and opioid pathways^64^. Several studies have reported that alcohol dependence was associated with increased risk of neuropsychiatric disorders, including bipolar disorder and autism^65–67^. Autism and alcohol dependence have been shown to share some genetic factors according to earlier studies, where a higher incidence of alcoholism in the family members of ASD patients compared with the general population was observed. Also, the links between the autism susceptibility candidate 2 gene (*AUTS2*), a known ASD risk gene, in the regulation of alcohol consumption was reported^67,68^. These findings support the hypothesis that *SERINC2* may be one of the dosage-sensitive genes and an increased SERINC2 protein may predispose to neurodevelopmental and neuropsychiatric disorders partly via abnormal NMD pathways and neurotransmitter or metabolic systems. In the DECIPHER database, we identified two CNVs of slightly larger size encompassing *SERINC2*; a 377.2-kb microdeletion that has been reported in a patient with seizure (patient 289527 in Supplementary Figure 1b), and a 434.6-kb microduplication reported in a patient with ID and short attention span who also has Potocki-Shaffer syndrome. While the microdeletion was deemed possibly pathogenic, the microduplication was inherited from healthy parent, raising the possibility of incomplete penetrance. In addition, two larger duplications containing *SERINC2* were seen in the ISCA/ClinGen database. These are a *de novo* 1.27-Mb duplication identified in a patient with global DD that was interpreted as pathogenic (nssv578518), and a 1.15-Mb VOUS identified in a patient with dysmorphic facial features (nssv1602204). Although the effects of other genes in these large duplication regions in the ISCA database could not be excluded, *SERINC2* is likely one of the susceptibility genes that may contribute to neuropsychiatric disorders as noted in the supportive evidence discussed above. Because this gene have not been previously linked to autism, we proposed the *SERINC2* gene as a novel ASD candidate gene. The supportive evidences of *SERINC2* as a potential ASD candidate gene are summarized in Supplementary Figure 4.

Alpha-thalassemia (α-thalassemia) is the most common incidental finding of CMA in our Thai population. In Northern Thailand and Laos, the prevalence of α-thalassemia has been estimated as between 30–40% including 3.6–10% of α-thalassemia 1 and 16.4–20% of α-thalassemia 2^69^. Since the majority of alterations in α-globin genes are deletions, a CMA with enough resolution should be able to detect these mutations. In this study, CMA testing could detect α-thalassemia in 9 of 114 ASD patients (7.89%); 4.39% α-thalassemia 1 (5/114), 1.75% α-thalassemia 2 (2/114), and 0.88% Hb H disease (1/114). A comparison of our CMA, which was a high resolution SNP array, and traditional single-tube multiplex PCR results indicated that CMA could accurately detect *HBA* gene deletion. In previous studies, oligonucleotide microarrays with custom design probes were successfully used to detect both α-thalassemia and β-thalassemia^70–72^. Thus, we suggest that a tentative diagnosis of α-thalassemia should be considered in patients who underwent CMA testing because this is important for genetic counseling and prevention of α-thalassemia, particularly in populations where the disease is endemic. Of note, distal deletion of chromosome 16p (16pter-16p13.3) causes alpha-thalassemia-intellectual deficit syndrome linked to chromosome 16 (ATR-16 syndrome, MIM 141750). Patients with ATR-16 syndrome present with either alpha-thalassemia trait or Hb H disease associated with mild to moderate ID, and with dysmorphic features in some cases. Deletion at 16p13.3 includes the loss of multiple genes including *HBA1* and *HBA2* resulting in alpha thalassemia phenotype, however the genes responsible for ID and other development abnormalities are not yet clearly identified. Some studies have suggested that deletion of *SOX8* at chromosome 16p13.3 could contribute to ID in patients with ATR-16 syndrome because of its high expression in the brain^73,74^. However, our ASD patients, who have alpha thalassemia trait due to 16p13.3 deletions ranging from 3,924–149,072 bp, were unlikely to have ATR-16 syndrome because the 16p13.3 deletions in previously reported patients were much larger (>800 kb)^74–76^ and included many other genes including *SOX8*.

We also reported an atypical PWS case with ASD caused by a UPD of chromosome 15. Although, the results of the microarray and MS-PCR analyses were consistent with Prader-Willi syndrome (PWS), the clinical features of this patient were milder than in typical PWS patients. Various studies have found that PWS patients with UPDs of 15q11-q13 had higher IQs, milder behavior problems^77,78^, and more pronounced clinical features of ASD than PWS patients with deletion of 15q11-q13^79^. Our patient carried two AOH regions at 15q11.1–15q22.2 and 15q26.1-15q26.3. The B-allele frequency (BAF) plot indicated both uniparental isodisomy and heterodisomy regions on chromosome 15, demonstrating results of the recombination process in meiosis (Supplementary Figure 1). From the BAF plot, we can also deduce that the non-disjunction occurred in maternal meiosis II, followed by post-zygotic loss of the paternal chromosome 15 by trisomy rescue^80^. Due to the many genes located within these two large AOH regions, there is a possibility that in addition to the maternal UPD15 there exists the interplay between multiple genes that may contribute to the atypical phenotype of this patient. However, more investigations are required to further elucidate the underlying pathogenesis.

In conclusion, our study is the first CMA testing in a large cohort of 114 patients with ASD from Thailand, a country which has to date been underrepresented in ASD studies. Our findings support the usefulness of CMA as a routine diagnostic test in patients with ASD of unknown cause. The CMA information can help not only medical management, but also in providing appropriate genetic counseling and evaluation of recurrence risk in families. In addition, we propose that the *SERINC2* gene is a novel ASD candidate gene. However, because of the limitation of the number of studies related to *SERINC2*, further investigations are required to confirm the effect of *SERINC2* in patients with ASD.

## Methods

### Patients

A total of 114 Thai patients with ASD of unknown cause were recruited in the study, 91 males and 23 females, with ages ranging from 1 to 18 years at the time of recruitment. They were divided into two cohorts. The first cohort (Group 1) was first evaluated for the common genetic causes of ASD and all patients, selected from a previous report^7^, had normal karyotype and negative DNA tests for Fragile X syndrome and *MECP2* mutations (defined as “non-syndromic ASD”). Therefore, they represented non-syndromic ASD patients with the use of CMA as a second line test. Group 1 patients comprised 68 retrospective cases with non-syndromic ASD, who met the DSM-IV criteria for ASD including 61 non-syndromic ASD with ID patients (nonverbal IQ < 70) and 7 non-syndromic ASD without ID patients (nonverbal IQ ≥ 70). The second cohort (Group 2) included 46 prospective ASD and DD cases with a clinical diagnosis of ASD based on the Diagnostic and Statistical Manual of Mental Disorders, Fifth Edition (DSM-5) criteria. DD is defined as performance at least two standard deviations below the age-appropriate mean and is not quantifiable with IQ testing. Group 2 patients were referred for CMA testing at Ramathibodi Hospital, Bangkok according to the American College of Medical Genetics and Genomics (ACMG) recommendations for the use of CMA as the first-tier diagnostic test in patients with unexplained ASD and DD.

The study was approved by the Institutional Ethics Committee of Faculty of Medicine, Prince of Songkla University (REC 48/364-006-3) and Faculty of Medicine Ramathibodi Hospital, Mahidol University (ID 12-57-03). The methods were carried out in accordance with the approved guidelines and relevant regulations. Written informed consent was obtained from the parents of each ASD individual.

### Chromosomal Microarray Analysis

CMA was performed with the Infinium CytoSNP-850K v1.1 Beadchip (Illumina, San Diego, California, USA), according to the manufacturer’s instructions. The array contains approximately 850,000 single nucleotide polymorphisms (SNPs) markers spanning the entire genome with an average probe spacing of 1.8 kb. The data were analyzed using BlueFuse Multi software v4.3 and GenomeStudio Data Analysis Software v. 2011.1 based on the reference human genome (hg19/GRCh37). Microarrays were carried out in available parents of the patients with pathogenic CNVs and VOUS to investigate the inheritance pattern in the families using either the Infinium CytoSNP-850K Beadchip (850,000 SNP markers) or HumanCytoSNP-12 DNA Analysis BeadChip v2.1 kit (~300,000 SNP markers) depending on the size of the interested CNVs.

### CNVs interpretation

In this study, only detected CNV with at least 10-probe coverage were reported. The detected CNVs were classified into pathogenic, benign, or variant of uncertain clinical significance (VOUS) according to established guidelines from the International Standard Cytogenomic Array (ISCA) and the ACMG^5,81^. The classification was based on the size of CNV, gene content, the inheritance pattern, and information in the medical literature and public databases. The Database of Genomic Variants (DGV) and the Thai CNV database (http://www.thaicnv.icbs.mahidol.ac.th/thaicnv/index.php)^34^, which contains CNVs from 3,017 Thai individuals, were used to exclude common structural variations in the Thai population, while the Online Mendelian Inheritance in Man (OMIM) database was used to identify disease-causing genes. The ISCA and Database of Chromosomal Imbalance and Phenotype in Humans using Ensembl Resources (DECIPHER) were used as reference for known microdeletion and microduplication syndromes. In addition, three ASD-related databases including the Simons Foundation for Autism Research Initiative (SFARI) (https://gene.sfari.org/autdb), the Autism DataBase (AUTDB) (http://autism.mindspec.org/autdb), and Autism Chromosome Rearrangement Database (ACRD; http://projects.tcag.ca/autism/) were utilized for interpretation of clinical significance.

CNVs overlapping with a region of known microdeletion or microduplication syndromes and/or disease-causing genes were classified as pathogenic. Even though a CNV was inherited from healthy parent, it could still be classified as pathogenic if incomplete penetrance was commonly observed. CNVs with insufficient information to determine whether they were pathogenic or benign were classified as VOUS. VOUS were further divided into likely pathogenic, likely benign and VOUS (no subclassification). CNVs were considered to be possibly pathogenic when they were *de novo* or inherited from an affected parent, and included genes associated with ID, ASD or neurological development or function. CNVs were considered to be possibly benign when they were inherited from a healthy parent and did not include genes known to be associated with a specific disease. CNVs were still considered to be VOUS with no subclassification when they contained genes with no or less known functions and had, to date, been considered as of unknown clinical significance in multiple publications and/or databases. CNVs that had been reported as common polymorphisms occurring in more than 1% of the population, and previously reported in multiple publications and normal population databases, were classified as benign and excluded from further analysis. Additionally, AOH regions larger than 10 Mb in size were also reported.

### Methylation-specific PCR (MS-PCR) for uniparental disomy detection

DNA samples were treated with sodium bisulfite, followed by *SNRPN* methylation specific PCR (MS-PCR). MS-PCR was performed with original primer sets designed by Kubota *et al*.^37^ and alternative primer sets designed by Hussain Askree *et al*.^36^. Untreated DNA was used as a control to ensure complete sodium bisulfate conversion of DNA, and amplified using primer set as previously described^36^. Subsequently, the PCR products were run on 2.5% agarose gel and visualized through ethidium bromide staining under a UV transilluminator.

### Statistical analysis

All statistical comparisons were made by Chi-square test using the online GraphPad QuickCalcs software (https://graphpad.com/quickcalcs/contingency1.cfm). An alpha level of 0.05 was used for all statistical analyses.

## Electronic supplementary material

Supplementary
