# Supplementary material for: Chromosomal microarray analysis in a cohort of underrepresented population identifies SERINC2 as a novel candidate gene for autism spectrum disorder
Source: Sci Rep. 2017 Sep 21;7:12096. doi: 10.1038/s41598-017-12317-3 (PMC5608768; doi:10.1038/s41598-017-12317-3)
Supplement: Supplementary file 1 — Supplementary [file 41598_2017_12317_MOESM1_ESM.pdf]

Supplementary

Chromosomal microarray analysis in a cohort of underrepresented population identifies *SERINC2* as a novel candidate gene for autism spectrum disorder

Areerat Hnoonual[1], Weerin Thammachote[2], Thipwimol Tim-Aroon[3], Kitiwan Rojnueangnit[4], Tippawan Hansakunachai[5], Tasanawat Sombuntham[6], Rawiwan Roongpraiwan[6], Juthamas Worachotekamjorn[7], Jariya Chuthapisith[6], Suthat Fucharoen[8], Duangrurdee Wattanasirichaigoon[3], Nichara Ruangdaraganon[6], Pornprot Limprasert[9,*], Natini Jinawath[2,10,*]

[1]Graduate Program in Biomedical Sciences, Prince of Songkla University, Songkhla, Thailand

[2]Program in Translational Medicine, Faculty of Medicine Ramathibodi Hospital, Mahidol University, Bangkok, Thailand

[3]Division of Medical Genetics, Department of Pediatrics, Faculty of Medicine Ramathibodi Hospital, Mahidol University, Bangkok, Thailand

[4]Division of Medical Genetics, Department of Pediatrics, Faculty of Medicine, Thammasart University, Pathumthani, Thailand

[5]Division of Child Development, Department of Pediatrics, Faculty of Medicine, Thammasart University, Pathumthani, Thailand

[6]Division of Developmental-Behavioral Pediatrics, Department of Pediatrics, Faculty of Medicine Ramathibodi Hospital, Mahidol University, Bangkok, Thailand

[7]Division of Child Development, Department of Pediatrics, Faculty of Medicine, Prince of Songkla University, Songkhla, Thailand

[8]Thalassemia Research Center, Institute of Molecular Biosciences, Mahidol University, Salaya, Nakhon Pathom, Thailand

[9]Division of Human Genetics, Department of Pathology, Faculty of Medicine, Prince of Songkla University, Songkhla, Thailand

[10]Integrative Computational Bioscience Center, Mahidol University, Salaya, Nakhon Pathom, Thailand

***Corresponding authors**

Pornprot Limprasert, MD, PhD

Division of Human Genetics,

Department of Pathology, Faculty of Medicine,

Prince of Songkla University, Hat Yai,

Songkhla 90110, Thailand

Tel: +66 74 451 584, Fax: +66 74 212 908

Email: lpornpro@medicine.psu.ac.th, lpornpro@yahoo.com

Natini Jinawath, MD, PhD

Program in Translational Medicine,

Faculty of Medicine Ramathibodi Hospital,

Mahidol University, Bangkok 10400, Thailand

Tel: +66 2 201 116, +66 2 201 2615, Fax +66 2 201 1611

Email: natini.jin@mahidol.ac.th, jnatini@hotmail.com

(a)

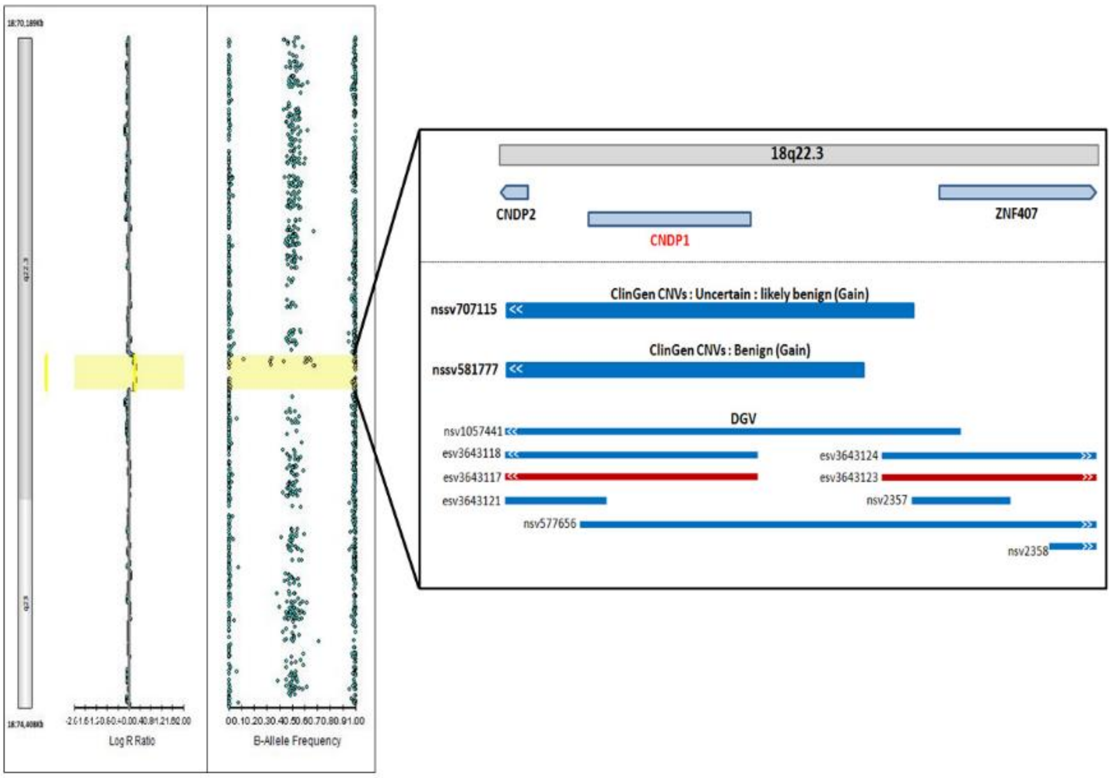
18q22.3
CNDP2
CNDP1
ZNF407
ClinGen CNVs : Uncertain : likely benign (Gain)
nssv707115
ClinGen CNVs : Benign (Gain)
nssv581777
DGV
nsv1057441
esv3643118
esv3643117
esv3643121
esv3643124
esv3643123
nsv2357
nsv577656
nsv2358
Log R Ratio
B-Allele Frequency

(b)

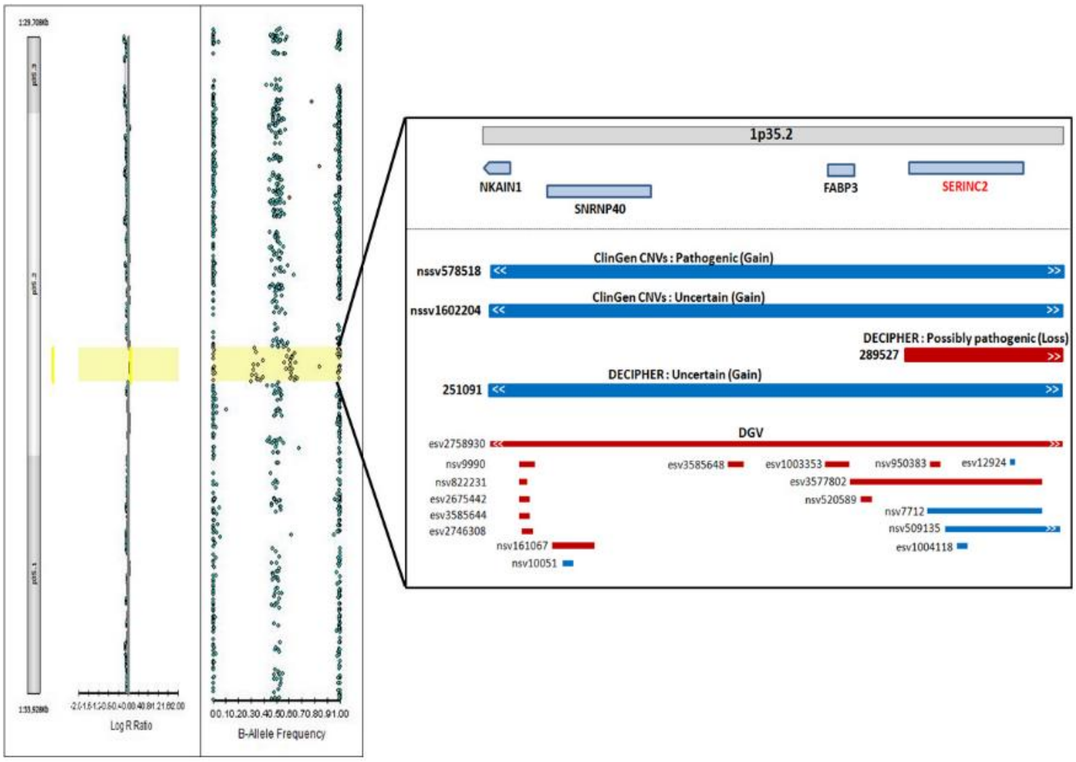
1p35.2
NKAIN1
SNRNP40
FABP3
SERINC2
ClinGen CNVs : Pathogenic (Gain)
nssv578518
ClinGen CNVs : Uncertain (Gain)
nssv1602204
DECIPHER : Possibly pathogenic (Loss)
289527
DECIPHER : Uncertain (Gain)
251091
DGV
esv2758930
nsv9990
nsv822231
esv2675442
esv3585644
esv2746308
nsv161067
nsv10051
esv3585648
esv1003353
nsv950383
esv12924
esv3577802
nsv520589
nsv7712
nsv509135
esv1004118
Log R Ratio
B-Allele Frequency

Supplementary Figure 1: Schematic representation of the CMA results of two *de novo* duplications. (a) a 217-kb duplication of 18q22.3 including *CNDP1* (patient AR12-3) and **(b)** a 220-kb duplication of 1p35.2 including *SERINC2* (patient TM41-3).The two duplications were mapped according to the UCSC genome browser (GRCh37/hg19 assembly; http://genome.ucsc.edu). Blue and red bars respectively represent duplications and deletions partially overlapped with the two CNVs. ClinGen CNVs in UCSC refer to the ISCA/ClinGen CNV database.

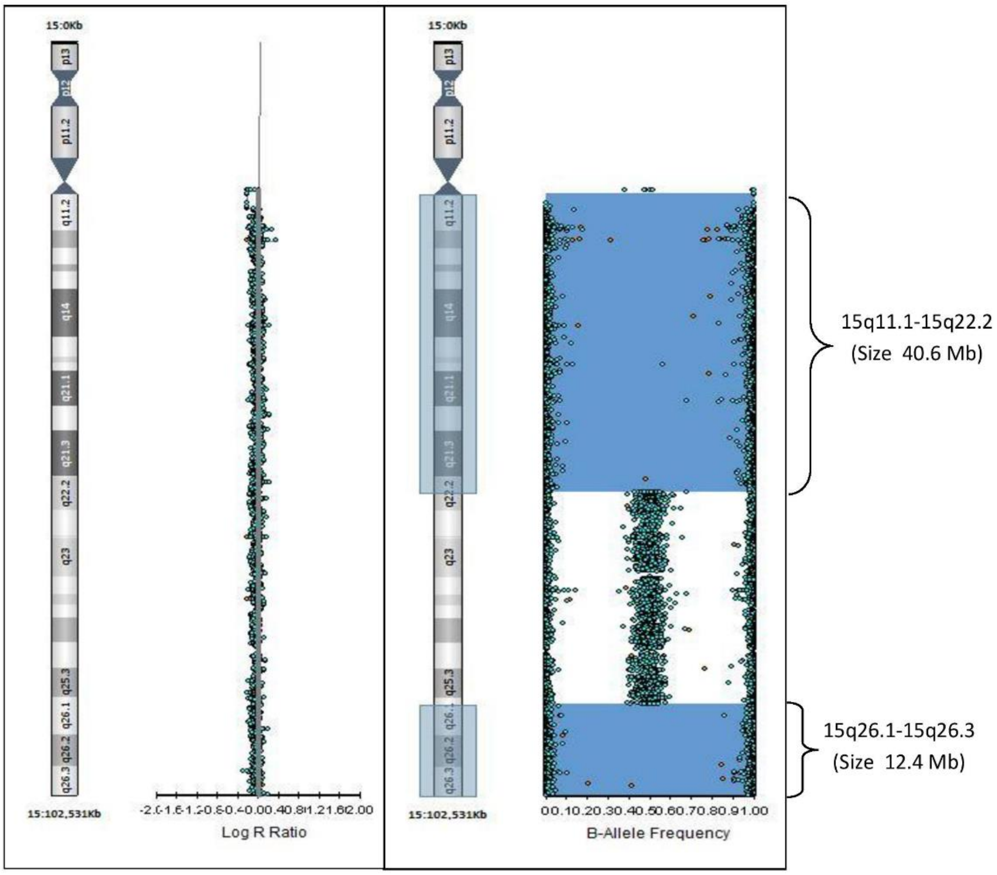

Supplementary Figure 2: The Log R ratio (LRR) (left) and B-allele frequency (BAF) (right) plots of chromosome 15 of an ASD patient showing two segments of uniparental isodisomy, a 40.6 Mb AOH at 15q11.1-15q22.2 and a 12.4 Mb AOH at 15q26.1-15q26.3.

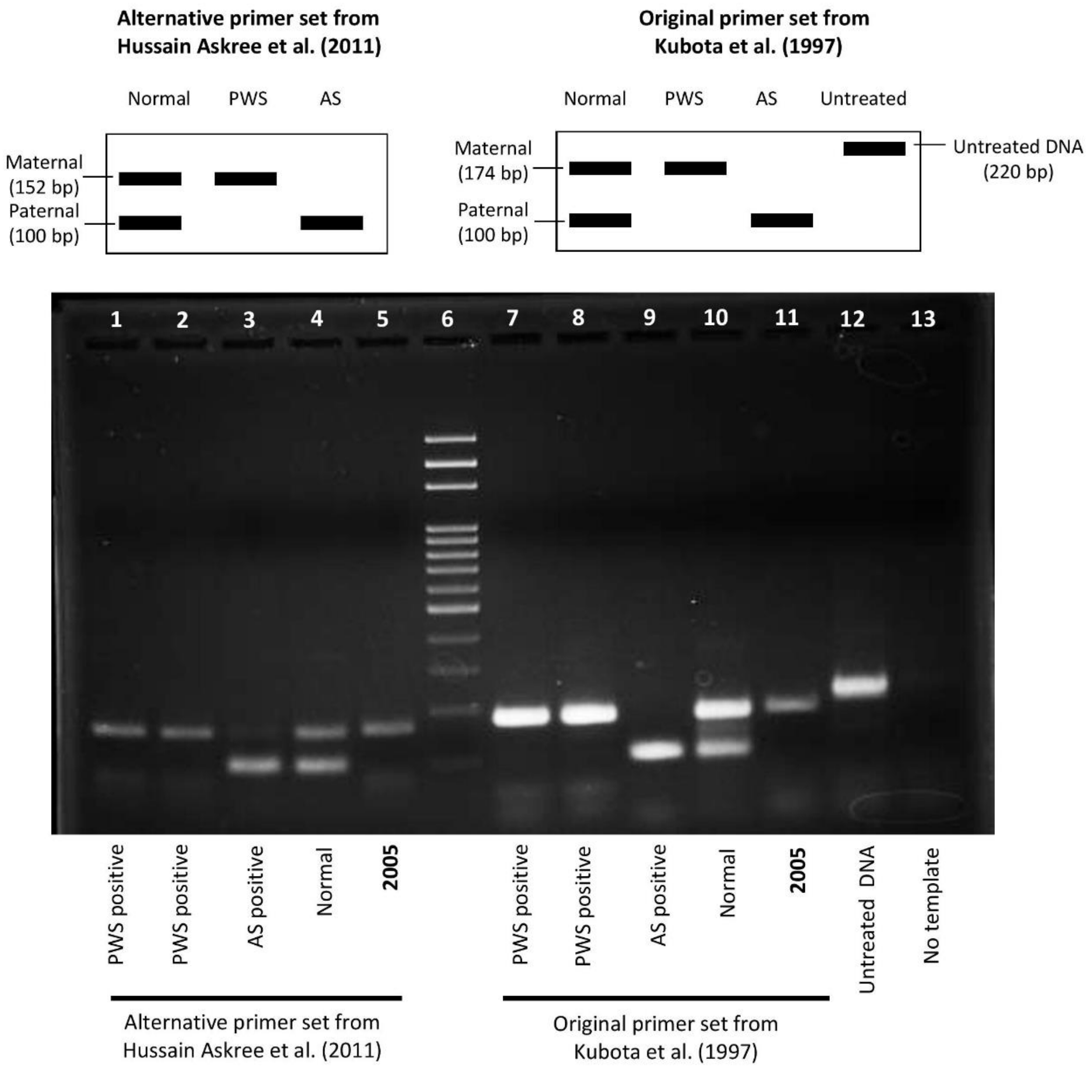

Supplementary Figure 3: MS-PCR analysis of patient (2005) with PWS. Upper panel represents the example of PCR products that were generated by MS-PCR analysis with original and alternative primer sets. **Lower panel** represents the results of MS-PCR analysis of patient (2005). Lane 1 to 5 represent MS-PCR results using alternative primer set designed by Hussain Askree et al.[1]: Lane 1 and 2, PWS positive controls; lane 3, AS positive control; lane 4, normal control; lane 5, patient sample (2005) showing only a maternally methylated allele. Lane 6 indicates 100-bp ladder. Lane 7 to 11 represent MS-PCR results using original primer set designed by Kubota et al.[2]: Lane 7 and 8, PWS positive controls; lane 9, AS positive control; lane 10, normal control; lane 11, patient sample (2005). Lane 12 indicates 220-bp product of untreated genomic DNA and lane 13 indicates no DNA template control. Abbreviations: PWS, Prader-Willi syndrome; AS, Angelman syndrome

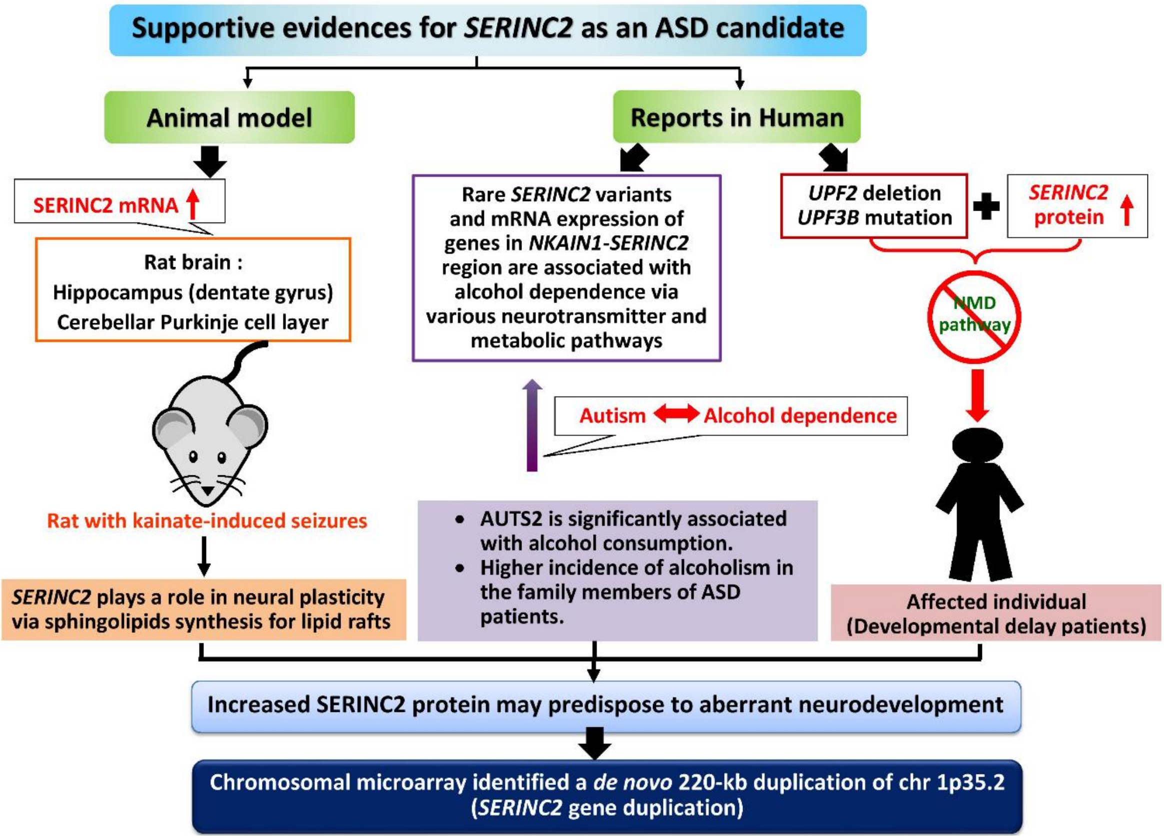

The mouse and person in Supplementary Figure 4 were created by A.H. and approved by P.L.

Supplementary Figure 4: The supportive evidences for *SERINC2* as an ASD candidate gene. Expression of *SERINC2* mRNA was upregulated in the dentate gyrus of the hippocampus and the cerebellar Purkinje cell layer from rats with kainate-induced seizures. The SERINC2 protein was upregulated in patients with developmental delay carrying the *UPF2* gene deletion or *UPF3B* gene mutation. In addition, rare *SERINC2* variants were significantly associated with alcohol dependence in the subjects of European descent. Transcript expressions of genes in *NKAIN1-SERINC2* genomic region were significantly associated with expressions of many genes in the neurotransmitter systems or metabolic pathways previously linked to alcohol dependence. Autism and alcohol dependence have been shown to share some genetic basis according to earlier studies. Therefore, *SERINC2* may be one of the dosage-sensitive genes and its duplication may predispose to neurodevelopmental and neuropsychiatric disorders partly via abnormal NMD pathways and neurotransmitter or metabolic pathways.

Case ID	CNVs	Size	Disease	OMIM gene involved
AR82-3	1q21.1-1q21.2 dup	1.25 Mb	1q21.1 duplication syndrome	***PRKAB2****, *FMO5, CHD1L,* ***BCL9****, *ACP6, GPR89A, GPR89B, GJA5,* ***GJA8****
TU22	4p16.3 del	1.95 Mb	Wolf-Hirschhorn syndrome	*ZNF141, PDE6B, ATP5I, MYL5, CPLX1, GAK, DGKQ, SLC26A1, IDUA, FGFRL1, RNF212, SPON2, CTBP1, MAEA, UVSSA, CRIPAK, SLBP, TACC3, FGFR3, LETM1, WHSC1, WHSC2*
2715	9q21.11-9q21.2 del	7.88 Mb	9q21.13 microdeletion syndrome	*APBA1, MAMDC2, SMC5, KLF9, MEMBER 3, MIR204, TMEM2, GDA, ZFAND5, TMC1, ALDH1A1, ANXA1,* ***RORB****, MEMBER 6, NMRK1, OSTF1,* ***PCSK5****, *RFK, GCNT1,* ***PRUNE2****, PCA3 ,VPS13A*
AR83-3	15q13.2-15q13.3 del	2.14 Mb	15q13.3 microdeletion syndrome	*CHRFAM7A, FAN1, TRPM1, MIR211, KLF13, OTUD7A, CHRFAM7A,* ***CHRNA7****
TM50-3	16p13.11 dup	1.50 Mb	16p13.11 microduplication syndrome	*PDXDC1,* ***NTAN1****, RRN3, NPIP, KIAA0430, ABCC1, ABCC6, NOMO3, NPIP, MYH11,* ***NDE1****
TU17	18q21.33-18q23 del	18.9 Mb	Distal 18q deletion syndrome	*CDH20, PIGN,TNFRSF11A, PHLPP, BCL2, FVT1, VPS4B, PI5, PI13, SERPINB4, SERPINB3, SERPINB7, SERPINB2, PI10, HMSD, PI8, CDH7, CDH19, DSEL, DOK6, CD226, RTTN, SOCS4, CBLN2, NETO1, FBXO15, TIMM21, CYB5A, FAM69C, PEPA, CNDP1,TSHZ1, ZNF516, ZNF236,* ***MBP****, *GALR1, SALL3, ATP9B, NFATC1, CTDP1, KCNG2, TXNL4A, PARD6G*
2950	22q13.33 del	673 kb	22q13.3 deletion syndrome (Phelan-McDermid Syndrome)	*MOV10L1, PANX2, SELENOO, TUBGCP6, HDAC10, MAPK12, MAPK11, PLXNB2, SAPS2, SBF1, ADM2, ALDRL6, NCAPH2, SCO2, TYMP, CPT1B, CHKB, MAPK8IP2, ARSA,* ***SHANK3****, *ACR, RABL2B, RABL2A*

Supplementary Table S1. Details of genes involved in pathogenic CNVs

* OMIM genes have been reported to be involved in neuropsychiatric disorders including ASD and ID.

No	Individual ID	Chromosome locus	Chromosome position (hg19)	Del (loss)/ Dup (gain)	Size (bp)	Genes involved	Confirmation by multiplex Gap-PCR
1	TM2-3	16p13.3	chr16:223477-227400	Loss	3,924	*HBA1* (exon1-part of exon3) & *HBA2* (exon 3)	α-thalassemia 2 (- $\alpha^{3.7}$/αα)
2	TM6-3	16p13.3	chr16:216742-233272	loss	16,530	*HBA1* & *HBA2*	α-thalassemia 1 (- -^{SEA}/ αα)
3	AR71-3	16p13.3	chr16:192314-341385	Loss	149,072	*HBA1* & *HBA2*	α-thalassemia 1 (- -^{SEA}/ αα)
4	TM50-3	16p13.3	chr16:216742-233272	loss	16,531	*HBA1* & *HBA2*	α-thalassemia 1 (- -^{SEA}/ αα)
5	RA29	16p13.3	chr16:216742-247888	Loss	31,147	*HBA1* & *HBA2*	α-thalassemia 1 (- -^{SEA}/ αα)
6	RA17	16p13.3	chr16:216742-233272	3 copies loss	16,531	*HBA1* & *HBA2*	Hb H disease (- $\alpha^{4.2}$/- -^{SEA})
7	2686	16p13.3	chr16:216742-233272	Loss	16,531	*HBA1* & *HBA2*	α-thalassemia 1 (- -^{SEA}/ αα)
8	2867	16p13.3	chr16:193586-233272	Loss	39,687	*HBA1* & *HBA2*	DNA not available
9	TU32	16p13.3	chr16:222140-227364	Loss	5,225	*HBA1* & *HBA2* (exon1-part of exon3)	α-thalassemia 2 (- $\alpha^{3.7}$/- $\alpha^{3.7}$)

Supplementary Table S2. Detection α-thalassemia in ASD patients by chromosomal microarray.

Author(s)	No. of patients	Location	Array platform	Yield of each type of CNV	Diagnostic yield (Data from publication)
**East Asian populations**					
Chong et al. (2014)[3]	105 ASD,ID/DD, MCA	China	Oligo (Agilent 44K and 180K)	Pathogenic CNVs : 19% (20/105 patients)<br>VOUS : 1.9% (2/105 patients)	Patients with pathogenic CNVs<br>**19%** (20/105 patients)
Tao et al. (2014)[4]	327 ASD,ID/DD, MCA	Hong Kong (China)	Oligo (NimbleGen CGX-135K array)	Pathogenic CNVs : 11.3% (27/327 patients)<br>VOUS : 12.2% (40/327 patients)	Patients with pathogenic CNVs<br>**11.3%** (37/327 patients)
	215 ASD,ID/DD **(Excluding 112 MCA, other)**			Pathogenic CNVs : 4.2% (9/215 patients)<br>VOUS : 13% (28/215 patients)	Patients with pathogenic CNVs<br>**4.2%** (9/215 patients)
Shin et al.(2015)[5]	96 ASD,ID/DD	Korea	Affymetrix CytoScan750K	Pathogenic CNVs : 15.6% (15/96 patients)<br>VOUS : 13.5% (13/96 patients)	Patients with pathogenic CNVs<br>**15.6%** (15/96 patients)
	42 ASD and ASD with ID/DD **(Excluding 54 ID/DD)**			Pathogenic CNVs : 0% (0/42 patients)<br>VOUS : 14.3% (6/42 patients)	Patients with pathogenic CNVs<br>**0% (0/42 patients)**
Siu et al. (2016)[6]	68 ASD	Hong Kong (China)	Roche NimbleGen CGX-135K	Pathogenic and likely pathogenic CNVs : 11.8% (8/68 patients)	Patients with Pathogenic and likely pathogenic CNVs<br>**11.8%** (8/68 patients)
This study	114 ASD	Thailand	Illumina, Infinium CytoSNP-850K	Pathogenic CNVs : 6.1% (7/114 patients)<br>VOUS : 19.3% (22/114 patients)	Patients with pathogenic CNVs and VOUS<br>**25.4%** (29/114 patients)

Supplementary Table S3: Continued

Author(s)	No. of patients	Location	Array platform	Yield of each type of CNV	Diagnostic yield (Data from publication)
**Middle East Asian populations**					
Al-Mamari et al. (2015)[7]	100 ASD	Oman	Oxford 8X60K	Pathogenic CNVs : 18% (18/100 patients)<br>VOUS : 9% (9/100 patients)	Patients with pathogenic CNVs and VOUS<br>**27%** (27/100 patients)
Soueid et al. (2016)[8]	41 ASD	Lebanon	Affymetrix Cytogenetics 2.7M and CytoScan HD	Pathogenic and likely pathogenic CNVs : 4.8% (2/41 patients)<br>VOUS : 29% (12/41 patients)	Patients with pathogenic and likely pathogenic CNVs<br>**4.8%** (2/41 patients)
**Non-Asian populations**					
Baldwin et al. (2008)[9]	211 ASD,ID/DD	USA	Oligo (Agilent 44K)	Pathogenic CNVs : 15.6% (33/211 patients)<br>VOUS : 2.4% (5/211 patients)	Patients with pathogenic CNVs<br>**15.6%** (33/211 patients)
Rosenfeld et al. (2010)[10]	1,461 ASD	USA	Oligo (Agilent 105K), BAC (SignatureChip v1-4 and SignatureChipWG)	Pathogenic CNVs : 7.7% (113/1,461 patients)	Patients with pathogenic CNVs<br>**7.7%** (113/1,461 patients)
Bremer et al. (2011)[11]	223 ASD	Sweden	BAC 33K and 38K arrays	Pathogenic CNVs : 8% (18/223 patients)<br>VOUS : 9% (20/223 patients)	Patients with pathogenic CNVs<br>**8%** (18/223 patients)
Iourov et al. (2012)[12]	54 ASD,ID/DD, MCA	Russia	BAC (Human BAC Array-System 12K)	Pathogenic CNVs : 28% (15/54 patients)	Patients with pathogenic CNVs<br>**28%** (15/54 patients)

Supplementary Table S3: Continued

Author(s)	No. of patients	Location	Array platform	Yield of each type of CNV	Diagnostic yield (Data from publication)
**Non-Asian populations *(Continued)***					
Shen et al. (2010)[13]	848 ASD	USA	SNP (Agilent CGH 244K, Affymetrix 500K v5)	Pathogenic and possibly pathogenic CNVs : 7.0% (59/848 patients)<br>VOUS : 11.2% (95/848 patients)	Patients with pathogenic and possibly pathogenic CNVs<br>**7.0%** (59/848 patients)
Coulter et al. (2011)[14]	1,792 ASD,ID/DD, MCA, other	USA	Not specified	Pathogenic CNVs : 7.3% (131/1,792 patients)<br>Possibly pathogenic CNVs : 5.8% (104/1,792 patients)	Patients with pathogenic and possibly pathogenic CNVs<br>**13.1%** (235/1,792 patients)
Ellison et al. (2012)[15]	46,298 ASD,ID/DD, MCA, other	USA	BAC-based arrays: 23,142 patients,<br>Oligo array (SignatureChip OS versions 1–3, Custom 135K): 23,156 patients	Pathogenic CNVs : 5.4% for oligo array<br>(1,259/23,156 patients)	Patients with pathogenic CNVs<br>**5.4%** for oligo array<br>(1,259/23,156 patients)
Filges et al. (2012)[16]	131 ASD, ID/DD	Switzer-land	SNP (NimbleGen 385K and 720K, Affymetrix Cytogenetics Whole-Genome 2.7 M array)	Pathogenic CNVs : 12.2% (16/131 patients)<br>VOUS : 12.2% (16/131 patients)	Patients with pathogenic CNVs<br>**12.2%** (16/131 patients)

Supplementary Table S3: Continued

Author(s)	No. of patients	Location	Array platform	Yield of each type of CNV	Diagnostic yield (Data from publication)
**Non-Asian populations *(Continued)***					
Battaglia et al.(2013)[17]	349 ASD,ID/DD	Italy	Oligo (Agilent 44K and 180K), SNP (Affymetrix v.6.0)	Pathogenic CNVs : 71.4% (65/91 CNVs)<br>Probably pathogenic CNVs : 8.8% (8/91 CNVs)<br>VOUS : 5.5% (5/91 CNVs)	Patients with pathogenic CNVs<br>**16.3%** (57/349 patients)
	78 ASD with ID/DD<br>**(Exclude 271 ID/DD)**			Pathogenic CNVs : 55% (11/20 CNVs)<br>Probably pathogenic CNVs : 20% (4/20 CNVs)<br>VOUS : 10% (2/10 CNVs)	Patients with pathogenic CNVs<br>**15.4%** (12/78 patients)
Sorte et al. (2013)[18]	50 ASD	Norway	Oligo (Agilent 105K)	Pathogenic CNVs : 16% (8/50 patients)	Patients with pathogenic CNVs<br>**16%** (8/50 patients)
Henderson et al. (2014)[19]	1,780 ASD, ID/DD, MCA, others	USA	Illumina HumanQuad610, HumanOmni1M	Pathogenic CNVs : 12.7% (227/1,780 patients)<br>VOUS : 13.5% (240/1,780 patients)	Patients with pathogenic CNVs<br>**12.7%** (227/1,780 patients)
Nava et al. (2014)[20]	194 ASD (from the 200 patients, six were excluded due to abnormal FISH, fragile X, RAI1 mutation results)	France	SNP (Illumina 370CNV-Quad ,660W-Quad, CytoSNP-12)	Pathogenic CNVs : 2.1% (4/194 patients)	Patients with pathogenic CNVs<br>**2.1%** (4/194 patients) excluding 4 patients with 16p11.2 microduplications)

Supplementary Table S3: Continued

Author(s)	No. of patients	Location	Array platform	Yield of each type of CNV	Diagnostic yield (Data from publication)
**Non-Asian populations *(Continued)***					
Nicholl et al. (2014)[21]	1,700 ASD,ID/DD, Epilepsy	Australia	Oligo (BlueGnome CytoChip ISCA 60K)	Pathogenic CNVs : 11.5% (195/1,700 patients)<br>VOUS : 11.5% (195/1,700 patients)	Patients with pathogenic CNVs<br>**11.5%** (195/1,700 patients)
	387 ASD, ASD with ID/DD<br>**(Excluding 1,313 ID/DD, Epilepsy)**			Pathogenic CNVs : 5.4% (21/387 patients)<br>VOUS : 9.8% (38/387 patients)	Patients with pathogenic CNVs<br>**5.4%** (21/387 patients)
Riggs et al. (2014)[22]	28,256 ASD, ID/DD,MCA, others	USA	Multiple platforms (not specified)	Pathogenic CNVs : 14.6% (4,125/28,256 patients)	Patients with pathogenic CNVs<br>**14.6%** (4,125/28,256 patients)
Roberts et al. (2014)[23]	215 ASD, learning disability	USA	Oligo (CombiMatrix 105K and 180K)	Pathogenic CNVs : 32 CNVs<br>VOUS : 17 CNVs	Patients with pathogenic CNVs and VOUS<br>**21%** (45/215 patients)
	65 ASD<br>**(Excluding 150 learning disability)**			Pathogenic CNVs : 6 CNVs<br>VOUS : 8 CNVs	Patients with pathogenic CNVs and VOUS<br>**20%** (13/65 patients)
Stobbe et al. (2014)[24]	23 ASD	USA	Oligo (NimbleGen 135K)	Pathogenic and likely pathogenic CNVs: 17.4% (4/23 patients)<br>VOUS : 22% (5/23 patients)	Patients with pathogenic and likely pathogenic CNVs<br>**17.4%** (4/23 patients)

Supplementary Table S3: Continued

Author(s)	No. of patients	Location	Array platform	Yield of each type of CNV	Diagnostic yield (Data from publication)
**Non-Asian populations *(Continued)***					
Eriksson et al. (2015)[25]	162 ASD	Sweden	Oligo (Agilent 244K and 180K, Oxford Gene Technology 180K)	Pathogenic CNVs : 8.6% (14/162 patients)<br>VOUS : 8.6% (14/162 patients)	Patients with pathogenic CNVs and VOUS<br>**17%** (28/162 patients)
Moreira et al. (2016)[26]	98 ASD	Brazil	Agilent Custom CGH 8X60K	Pathogenic CNVs : 9.2% (9/98 patients)	Patients with pathogenic CNVs<br>**9.2%** (9/98 patients)
Oikonomakis et al. (2016)[27]	195 ASD	Greece	Agilent 244K, 4X180K, 4X180K CGH+SNP	Pathogenic CNVs : 78.5% (51/65 CNVs)<br>VOUS : 21.5% (14/65 CNVs)	Patients with pathogenic CNVs and VOUS<br>**26.1%** (51/195 patients)
Xu et al. (2016)[28]	115ASD,ID/DD	USA	BlueGenome CytoChip v2 CGH, Affymetrix Cytogenetics v6.0 and CytoScan HD	Pathogenic CNVs : 22 CNVs<br>Likely pathogenic CNVs : 5 CNVs<br>VOUS : 22 CNVs	Patients with pathogenic CNVs<br>**18.3%** (21/115 patients)

Supplementary Table S3: Summary of diagnostic yields of chromosomal microarray in ASD patients in our and previous studies. Abbreviations: VOUS, variant of uncertain clinical significance; ASD, autism spectrum disorder; ID, intellectual disability; DD, developmental delay; MCA, multiple congenital anomalies; Autism Genetic Resource Exchange (AGRE); CGH, comparative genomic hybridization; BAC, bacterial artificial chromosome

References

1 Hussain Askree, S. *et al.* Allelic dropout can cause false-positive results for Prader-Willi and Angelman syndrome testing. *J Mol Diagn*. **13**, 108-112 (2011).

2 Kubota, T. *et al.* Methylation-specific PCR simplifies imprinting analysis. *Nat Genet*. **16**, 16-17 (1997).

3 Chong, W. W. *et al.* Performance of chromosomal microarray for patients with intellectual disabilities/developmental delay, autism, and multiple congenital anomalies in a Chinese cohort. *Mol Cytogenet*. **7**, 34 (2014).

4 Tao, V. Q. *et al.* The clinical impact of chromosomal microarray on paediatric care in Hong Kong. *PLoS One*. **9**, e109629 (2014).

5 Shin, S., Yu, N., Choi, J. R., Jeong, S. & Lee, K. A. Routine chromosomal microarray analysis is necessary in Korean patients with unexplained developmental delay/mental retardation/autism spectrum disorder. *Ann Lab Med*. **35**, 510-518 (2015).

6 Siu, W. K. *et al.* Diagnostic yield of array CGH in patients with autism spectrum disorder in Hong Kong. *Clin Transl Med*. **5**, 18 (2016).

7 Al-Mamari, W. *et al.* Diagnostic Yield of Chromosomal Microarray Analysis in a Cohort of Patients with Autism Spectrum Disorders from a Highly Consanguineous Population. *J Autism Dev Disord*. **45**, 2323-2328 (2015).

8 Soueid, J. *et al.* RYR2, PTDSS1 and AREG genes are implicated in a Lebanese population-based study of copy number variation in autism. *Sci Rep*. **6**, 19088 (2016).

9 Baldwin, E. L. *et al.* Enhanced detection of clinically relevant genomic imbalances using a targeted plus whole genome oligonucleotide microarray. *Genet Med*. **10**, 415-429 (2008).

10 Rosenfeld, J. A. *et al.* Copy number variations associated with autism spectrum disorders contribute to a spectrum of neurodevelopmental disorders. *Genet Med*. **12**, 694-702 (2010).

11 Bremer, A. *et al.* Copy number variation characteristics in subpopulations of patients with autism spectrum disorders. *Am J Med Genet B Neuropsychiatr Genet*. **156**, 115-124 (2011).

12 Iourov, I. Y. *et al.* Molecular karyotyping by array CGH in a Russian cohort of children with intellectual disability, autism, epilepsy and congenital anomalies. *Mol Cytogenet*. **5**, 46 (2012).

13 Shen, Y. *et al.* Clinical genetic testing for patients with autism spectrum disorders. *Pediatrics*. **125**, e727-735 (2010).

14 Coulter, M. E. *et al.* Chromosomal microarray testing influences medical management. *Genet Med*. **13**, 770-776 (2011).

15 Ellison, J. W. *et al.* Clinical Utility of Chromosomal Microarray Analysis. *Pediatrics*. **130**, e1085-e1095 (2012).

16 Filges, I. *et al.* High resolution array in the clinical approach to chromosomal phenotypes. *Gene*. **495**, 163-169 (2012).

17 Battaglia, A. *et al.* Confirmation of chromosomal microarray as a first-tier clinical diagnostic test for individuals with developmental delay, intellectual disability, autism spectrum disorders and dysmorphic features. *Eur J Paediatr Neurol*. **17**, 589-599 (2013).

18 Sorte, H. S., Gjevik, E., Sponheim, E., Eiklid, K. L. & Rodningen, O. K. Copy number variation findings among 50 children and adolescents with autism spectrum disorder. *Psychiatr Genet*. **23**, 61-69 (2013).

19 Henderson, L. B. *et al.* The impact of chromosomal microarray on clinical management: a retrospective analysis. *Genet Med*. **16**, 657-664 (2014).

20 Nava, C. *et al.* Prospective diagnostic analysis of copy number variants using SNP microarrays in individuals with autism spectrum disorders. *Eur J Hum Genet*. **22**, 71-78 (2014).

21 Nicholl, J. *et al.* Cognitive deficit and autism spectrum disorders: prospective diagnosis by array CGH. *Pathology*. **46**, 41-45 (2014).

22 Riggs, E. R. *et al.* Chromosomal microarray impacts clinical management. *Clin Genet*. **85**, 147-153 (2014).

23 Roberts, J. L., Hovanes, K., Dasouki, M., Manzardo, A. M. & Butler, M. G. Chromosomal microarray analysis of consecutive individuals with autism spectrum disorders or learning disability presenting for genetic services. *Gene*. **535**, 70-78 (2014).

24 Stobbe, G. *et al.* Diagnostic yield of array comparative genomic hybridization in adults with autism spectrum disorders. *Genet Med*. **16**, 70-77 (2014).

25 Eriksson, M. A. *et al.* Rare copy number variants are common in young children with autism spectrum disorder. *Acta Paediatr*. **104**, 610-618 (2015).

26 Moreiraa, E. S. *et al.* Detection of small copy number variations (CNVs) in autism spectrum disorder (ASD) by custom array comparative genomic hybridization (aCGH). *Research in Autism Spectrum Disorders*. **23**, 145–151 (2016).

27 Oikonomakis, V. *et al.* Recurrent copy number variations as risk factors for autism spectrum disorders: analysis of the clinical implications. *Clin Genet*. **89**, 708-718 (2016).

28 Xu, Q. *et al.* Chromosomal microarray analysis in clinical evaluation of neurodevelopmental disorders-reporting a novel deletion of SETDB1 and illustration of counseling challenge. *Pediatr Res*. **80**, 371-381 (2016).
